# Supplementary material for: Australian Women Veterans’ Experiences of Gendered Disempowerment and Abuse Within Military Service and Transition
Source: Int J Environ Res Public Health. 2025 Apr 8;22(4):584. doi: 10.3390/ijerph22040584 (PMC12026488; doi:10.3390/ijerph22040584)
Supplement: Supplementary file 1 [file ijerph-22-00584-s001.zip › ijerph-3442126-supplementary.pdf]

Supplementary File S1: Chronology of Women's Involvement in the Australian Military

Historically, the **ADF was a male-dominated institution**, but significant changes have occurred over the past century, particularly regarding the inclusion of women.

Women in the ADF – A Timeline

- **World War I (1914–1918):** Women served as nurses in the Australian Army Nursing Service (AANS) but were not allowed to engage in combat roles.
- **World War II (1939–1945):** Women were allowed to enlist in auxiliary roles such as communications, logistics, and intelligence. The Women's Royal Australian Naval Service (WRANS) and Women's Auxiliary Australian Air Force (WAAAF) were established.
- **Post-War Period (1945–1970s):** Women's participation remained limited, mostly in administrative and healthcare roles.
- **1975 – Australia Ratifies the UN Convention on the Elimination of All Forms of Discrimination Against Women (CEDAW):** This set the stage for broader gender integration in the ADF.
- **1980s – Expansion of Roles:** Women were gradually allowed to serve in non-combat roles across the three branches of the ADF.
- **1992 – Lifting of Combat Exclusion:** Women could serve in combat-related but not front-line roles.
- **2011 – Full Combat Inclusion:** The Australian government removed all gender-based restrictions, allowing women to serve in any military role, including special forces.
- **2020s – Women's Representation:** Today, women make up about **20% of the ADF**, with increasing representation in leadership roles.

Gender Challenges in the ADF

Despite progress, challenges remain for women in the ADF, including:

- **Military Culture and Gender Bias:** Reports of sexual harassment and discrimination persist, as highlighted in various **Defence Force Reviews and Royal Commissions**.
- **Work-Life Balance:** The demands of military service pose challenges for women, particularly regarding **family and parental responsibilities**.
- **Retention and Career Progression:** Women face barriers in promotions, especially in combat-intensive roles.

Gender Reforms in the ADF

To address these challenges, the ADF has introduced reforms such as:

- **The Pathway to Change Strategy:** Focuses on inclusivity and diversity within the ADF.
- **Sex Discrimination Commissioner Reviews (2011–2014):** Recommended measures to improve gender equality.
- **Family-Friendly Policies:** Including **flexible work arrangements** and **parental leave support**.

Challenges and Reforms

Despite progress, women in the ADF have faced challenges, including instances of sexual harassment and systemic biases. The Review into the Treatment of Women in the Australian Defence Force highlighted the necessity for cultural and structural reforms to address these issues.

Sources:

[1] Australian Defence Force. *Defence Annual Report 2023–2024*; Australian Government: Canberra, Australia, 2024. Available online: https://www.defence.gov.au/about/accessing-information/annual-reports (accessed on 27 February 2025).

[11] Department of Defence. *Women in the Australian Defence Force (ADF) 2021–2022 Ten Years in Review*; Commonwealth of Australia: Canberra, Australia, 2023. Available online: https://www.defence.gov.au/sites/default/files/2023-08/women-in-adf-2021-22.pdf (accessed on 27 February 2025).

[65] Reghenzani, C. *Women in the ADF: six decades of policy change (1950 to 2011).* Summer Scholars Paper for the Department of Parliamentary Services, Australian Government, Canberra, 2015. https://www.aph.gov.au/-/media/05_About_Parliament/54_Parliamentary_Depts/544_Parliamentary_Library/pubs/apf/scholarship/CReghenzani.pdf?la=en&hash=C676B906A183BF93A4A403474AAAA117C84905DE (accessed on 27 February 2025).
